# Dietary Protein Intake in Dutch Elderly People: A Focus on Protein Sources

**DOI:** 10.3390/nu7125496

**Published:** 2015-11-25

**Authors:** Michael Tieland, Karin J. Borgonjen-Van den Berg, Luc J. C. Van Loon, Lisette C. P. G. M. de Groot

**Affiliations:** 1 Top Institute Food and Nutrition, P.O. Box 557, Wageningen 6700 AN, The Netherlands; michael.tieland@wur.nl (M.T.); l.vanloon@maastrichtuniversity.nl (L.J.C.V.L.); 2 Division of Human Nutrition, Wageningen University, P.O. Box 17, Wageningen 6700 AA, The Netherlands; karin.borgonjen@wur.nl; 3 Department of Human Movement Sciences, NUTRIM School for Nutrition, Toxicology and Metabolism, Maastricht University Medical Centre+, P.O. Box 616, Maastricht 6200 MD, The Netherlands

**Keywords:** protein, amino acids, aging, food sources, malnutrition

## Abstract

Introduction: Sufficient high quality dietary protein intake is required to prevent or treat sarcopenia in elderly people. Therefore, the intake of specific protein sources as well as their timing of intake are important to improve dietary protein intake in elderly people. Objectives: to assess the consumption of protein sources as well as the distribution of protein sources over the day in community-dwelling, frail and institutionalized elderly people. Methods: Habitual dietary intake was evaluated using 2- and 3-day food records collected from various studies involving 739 community-dwelling, 321 frail and 219 institutionalized elderly people. Results: Daily protein intake averaged 71 ± 18 g/day in community-dwelling, 71 ± 20 g/day in frail and 58 ± 16 g/day in institutionalized elderly people and accounted for 16% ± 3%, 16% ± 3% and 17% ± 3% of their energy intake, respectively. Dietary protein intake ranged from 10 to 12 g at breakfast, 15 to 23 g at lunch and 24 to 31 g at dinner contributing together over 80% of daily protein intake. The majority of dietary protein consumed originated from animal sources (≥60%) with meat and dairy as dominant sources. Thus, 40% of the protein intake in community-dwelling, 37% in frail and 29% in institutionalized elderly originated from plant based protein sources with bread as the principle source. Plant based proteins contributed for >50% of protein intake at breakfast and between 34% and 37% at lunch, with bread as the main source. During dinner, >70% of the protein intake originated from animal protein, with meat as the dominant source. Conclusion: Daily protein intake in these older populations is mainly (>80%) provided by the three main meals, with most protein consumed during dinner. More than 60% of daily protein intake consumed is of animal origin, with plant based protein sources representing nearly 40% of total protein consumed. During dinner, >70% of the protein intake originated from animal protein, while during breakfast and lunch a large proportion of protein is derived from plant based protein sources.

## 1. Introduction

Sarcopenia, the age related loss of skeletal muscle mass and strength, is accompanied by a decline in functional ability that affects many aspects of life [1]. Sarcopenia is a process caused by a combination of factors, which include a more sedentary lifestyle and an inadequate dietary protein intake [2,3]. It has been well-established that dietary protein ingestion stimulates skeletal muscle protein synthesis [4,5,6,7,8] and inhibits protein breakdown, resulting in a positive protein balance [7,8] and net muscle mass gain [9,10,11]. However, with the ingestion of small, meal-like amounts of dietary protein, the postprandial skeletal muscle protein synthetic response is attenuated in elderly people [12,13]. Such an attenuated post-prandial muscle protein synthetic response, also referred as “anabolic resistance,” would result in a negative skeletal muscle protein balance, and subsequent loss of skeletal muscle mass. To overcome the anabolic resistance to small protein intakes, it has been suggested that 25 to 30 g of dietary protein per meal is required to allow an appropriate stimulation of postprandial muscle protein synthesis in the elderly. Further to the level of protein intake, protein sources and the timing of their intake are considered important. Even though all protein sources have the capacity to stimulate muscle protein synthesis, postprandial muscle protein fractional synthetic rates can vary substantially following the ingestion of different protein sources [14]. Insight in the intake and timing of dietary protein sources would yield valuable information to locate protein intake inadequacies and to develop effective countermeasures to further optimize muscle protein synthesis in the elderly. However, data presenting the intake of specific protein containing food sources as well as the timing of intake of those protein sources in elderly people is limited. Therefore, we aim to examine the consumption of protein sources as well as the distribution of protein sources over the day in community-dwelling, frail and institutionalized elderly people.

## 2. Methods

### 2.1. Study Population

We used data from community-dwelling, frail and institutionalized elderly people. Data on the community-dwelling elderly were derived from the most recent Dutch National Food Consumption Survey (DNFCS) conducted in 2011 [15]. Dietary intake data for 739 independently living elderly men and women were collected using two non-consecutive dietary records assisted with 24 h recalls. Data for frail elderly people were derived from baseline dietary assessments of two randomized placebo-controlled trials [16,17,18]. Dietary intake data on 321 frail elderly men and women were collected by trained dieticians using a three-day food record conducted on two non-consecutive weekdays and one weekend day [16,17,18]. The criteria for frailty used are descripted elsewhere and mainly focus on the physical aspects of frailty [19,20]. Data of the institutionalized elderly people were derived from the baseline dietary assessment of a large trial performed in Dutch nursing home residents. Dietary intake data were collected from 219 elderly people housed in somatic wards using three-day food records. Observations and a weighing-back method were applied to increase the validity of the dietary intake assessment [21]. In addition to the dietary intake data, subject characteristics including age, sex, physical activity, body weight, and BMI were used if available.

### 2.2. Calculation of Dietary Protein Intake

Trained dieticians gave oral and written instructions regarding documentation of food sources, estimation of the portion size using household measures and the timing of intake. Dieticians cross-checked the records for completeness and obtained additional information to optimize dietary intake assessment. Dietary intake data were coded (type of food, time of intake and amount) and energy and protein were calculated using Compl-eat (version 1.0, Wageningen University, Wageningen, The Netherlands) and the Dutch national food composition database of 2011. Protein-containing food sources were assessed and presented as a percentage of the total protein, animal and vegetable intake. In addition, the top five protein-containing food sources were assessed per meal and presented as percentage of total protein intake of that meal.

### 2.3. Statistical Analysis

Data analyses were performed using SPSS Statistics (version 21, IBM, Armonk, NY, USA). Data were presented as mean ± standard deviation or percentage. Pearson’s correlation coefficients were calculated to assess the relation of energy and protein intake in the elderly.

## 3. Results

**Figure 1:**
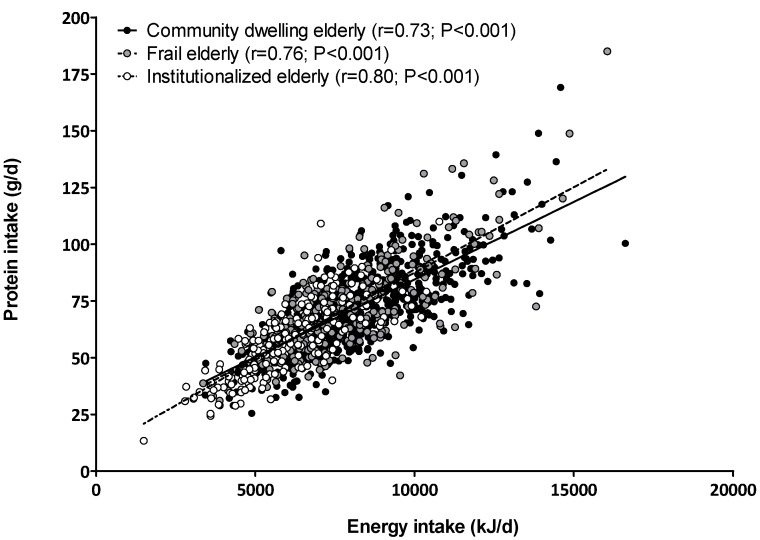
Scatterplot of dietary energy and protein intake in community-dwelling, frail and institutionalized elderly people.

Descriptive characteristics and dietary intake data of the study populations are presented in Table 1. The mean age was 77 ± 5, 79 ± 7 and 80 ± 8 years and there were 50%, 45% and 62% women in the community-dwelling, frail and institutionalized elderly population, respectively. Daily energy intake was higher in community-dwelling elderly (8097 ± 1953 kJ/day) when compared with frail (7753 ± 2012 kJ/day) and institutionalized elderly people (6140 ± 1638 kJ/day). Likewise, daily protein intake was 71 ± 18 g/day in community-dwelling, 71 ± 20 g/day in frail and 58 ± 16 g/day in institutionalized elderly people and accounted for 16% ± 3%, 16% ± 3% and 17% ± 3% of their energy intake, respectively. The intake of dietary protein strongly correlates with energy intake in community-dwelling (*r* = 0.73 CI: 0.70–0.77), frail (*r* = 0.76 CI: 0.71–0.80) and institutionalized (*r* = 0.80 CI: 0.75–0.84) elderly people (Figure 1).

**Table 1:** Subject characteristics and nutritional intake of community-dwelling, frail and institutionalized elderly people.

Subject Characteristics	Community-Dwelling Elderly (*n* = 739)		Frail Elderly (*n* = 321)		Institutionalized Elderly (*n* = 219)	
	Mean	SD	Mean	SD	Mean	SD
Age (year)	77.2	5.2	78.5	6.5	80.2	7.7
Women	77.6	5.4	77.9	6.1	81.2	7.9
Men	76.7	5.0	79.8	7.1	78.8	7.1
Sex, women %	50	-	45	-	62	-
Weight (kg)	77.2	12.4	70.1	12.4	72.0	18.3
Women	71.6	11.3	66.9	11.6	71.5	17.3
Men	82.7	10.9	77.2	11.2	72.8	20.0
Height (m)	1.67	0.09	1.65	0.09	1.62	0.11
Women	1.61	0.06	1.61	0.06	1.57	0.09
Men	1.74	0.06	1.73	0.07	1.69	0.09
BMI (kg/m^2^)	27.4	3.8	25.7	3.8	27.9	5.7
Women	27.6	4.3	25.7	4.0	28.8	6.3
Men	27.2	3.2	25.5	3.1	26.2	4.0
Nutritional intake						
Energy intake (kJ/day)	8089	1944	7749	2010	6148	1635
Women	7230	1646	7178	1654	5943	1604
Men	8932	1844	8992	2159	6471	1640
Protein intake (g/day)	71	18	71	20	58	16
Women	65	16	66	16	55	15
Men	78	18	80	24	63	18
Protein intake (en%)	15	3	16	3	16	3
Women	15	3	16	3	16	3
Men	15	3	15	3	16	3
Protein intake (g/kg-bw/day)	0.9	0.3	1.0	0.3	0.8	0.3
Women	0.9	0.3	1.0	0.3	0.8	0.3
Men	0.9	0.2	1.0	0.3	0.9	0.3
Plant protein (g)	28	9	25	7	16	6
Women	25	7	23	6	15	5
Men	30	9	29	9	17	6
Plant protein (%)	40	10	37	9	28	8
Women	40	10	37	9	28	9
Men	40	9	37	9	29	8
Animal protein (g)	44	14	46	17	42	14
Women	40	13	43	14	40	12
Men	47	14	52	20	45	16
Animal protein (%)	60	10	63	9	71	9
Women	60	10	63	9	72	8
Men	60	9	63	9	70	10

Values are means ± SD; BMI: body mass index; en%: energy percentage; g/kg-bw/day: gram per kilogram bodyweight per day.

Table 2 shows the distribution of dietary protein intake throughout the day in community-dwelling, frail and institutionalized elderly people. Dietary protein intake at breakfast was 11.5 ± 6.5 g in community-dwelling, 10.3 ± 6.2 g in frail and 12.3 ± 6.1 g in institutionalized elderly people. Breakfast contributed to 15%–21% of daily protein intake. More protein was ingested at lunch (from 15 g in institutionalized elderly to 23 g in community-dwelling elderly people) and dinner (from 24 g in institutionalized elderly to 31 g in frail elderly people), which contributed 26%–32% and 38%–44% to the daily protein intake, respectively. Protein intake at breakfast, lunch and dinner contributed >80% of total protein intake.

**Figure 2:**
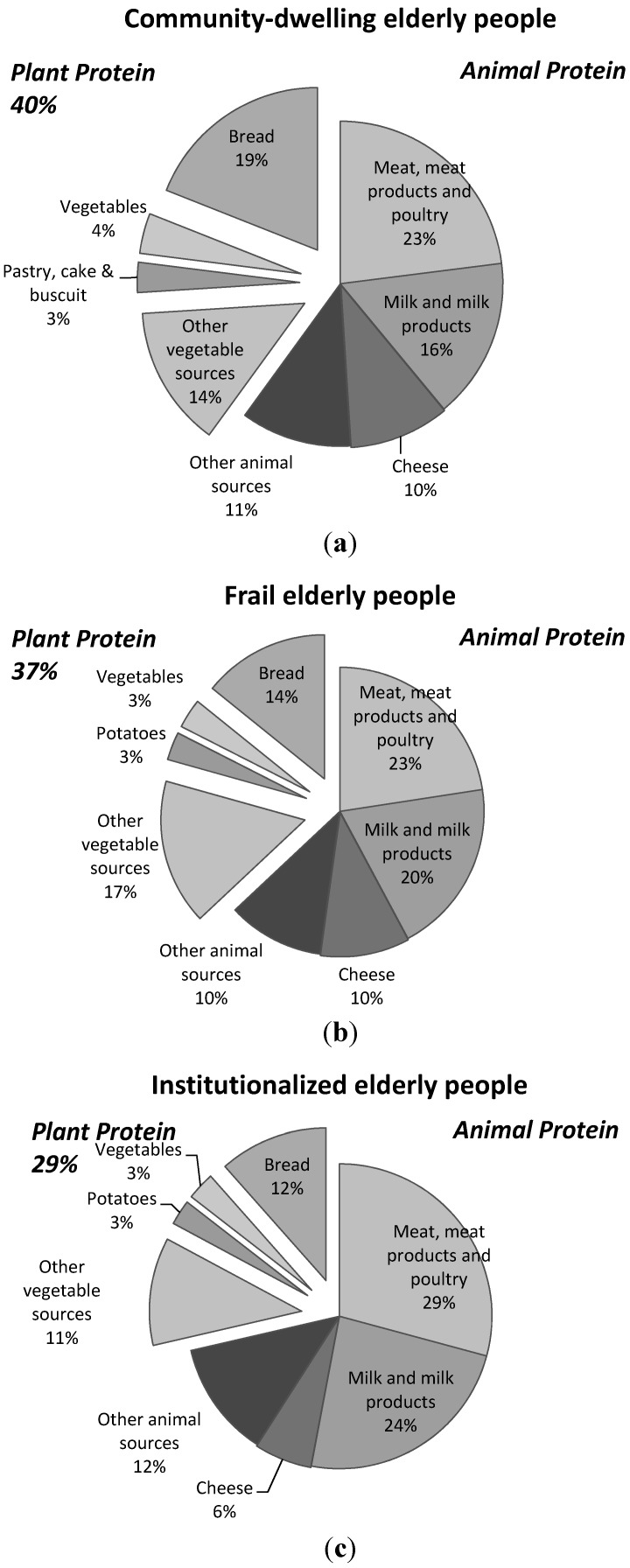
Contribution of plant and animal protein sources to total daily protein intake in community-dwelling (**a**); frail (**b**) and institutionalized elderly people (**c**).

Figure 2 presents the distribution of animal and plant based protein sources in community-dwelling, frail and institutionalized elderly people. Of the total protein intake, 40%, 37% and 29% originated from plant proteins in community-dwelling, frail, and institutionalized elderly people, respectively, with bread as the dominant source. The most dominant animal based protein sources were meat and dairy, which together accounted for 39%–53% of the total protein intake (Figure 2).

In both the community-dwelling and frail elderly, >50% of their protein intake at breakfast was derived from plant-based protein sources (Table 2) with bread (44% in community-dwelling and 39% in frail elderly) as the dominant source (Table 3). In contrast, the majority of protein intake at breakfast in institutionalized elderly people was derived from animal sources, with dairy (39%) as major protein source (Table 3). During lunch, 34%–37% derived from plant-based protein sources. Bread contributed the most with 23%–30% of the total protein intake at lunch. The main animal-based protein sources during lunch were meat and dairy products. During dinner, >70% of the protein intake originated from animal-based protein sources. In particular, meat products (36% in community-dwelling and 44% in frail and institutionalized elderly) contributed to protein intake at dinner.

**Table 2:** Dietary protein intake of community-dwelling, frail and institutionalized elderly people distributed throughout the day.

Protein Intake	Breakfast			Morning Snack			Lunch			Afternoon Snack			Dinner			Evening Snack		
	CD	Frail	INST	CD	Frail	INST	CD	Frail	INST	CD	Frail	INST	CD	Frail	INST	CD	Frail	INST
Protein intake (g/day)	11 (7)	10 (7)	12 (7)	3 (4)	3 (4)	2 (3)	22 (13)	18 (10)	15 (8)	4 (6)	4 (6)	3 (4)	27 (13)	31 (15)	24 (12)	5 (6)	5 (7)	2 (4)
Protein intake (% of total protein intake)	16%	14%	21%	4%	5%	3%	31%	26%	25%	5%	5%	6%	38%	43%	41%	7%	7%	4%
Plant-based protein (g/day)	6 (4)	5 (3)	4 (2)	1 (2)	1 (2)	1 (2)	8 (4)	7 (4)	5 (3)	2 (2)	2 (3)	1 (1)	8 (5)	8 (4)	5 (3)	2 (3)	2 (3)	1 (1)
Animal protein (g/day)	5 (5)	5 (6)	8 (6)	1 (2)	2 (3)	1 (2)	14 (11)	11 (9)	10 (7)	2 (5)	2 (5)	2 (4)	19 (13)	23 (14)	19 (11)	3 (5)	3 (5)	1 (3)

Values are means ± SD; CD: Community-dwelling elderly; INST: Institutionalized elderly.

**Table 3:** Top 5 dietary protein sources during breakfast, lunch, dinner and snacks in community-dwelling, frail and institutionalized elderly people.

Breakfast			Morning Snack			Lunch			Afternoon Snack			Dinner			Evening Snack		
CD	Frail	INST	CD	Frail	INST	CD	Frail	INST	CD	Frail	INST	CD	Frail	INST	CD	Frail	INST
Bread (44%)	Bread (38%)	Milk and milk products (40%)	Milk and milk products (29%)	Milk and milk products (49%)	Milk and milk products (50%)	Meat, meat products and poultry (25%)	Bread (30%)	Milk and milk products (31%)	Pastry, cake and biscuits (19%)	Milk and milk products (27%)	Milk and milk products (38%)	Meat, meat products and poultry (36%)	Meat, meat products and poultry (44%)	Meat, meat products and poultry (44%)	Milk and milk products (30%)	Milk and milk products (37%)	Milk and milk products (53%)
Cheese (18%)	Milk and milk products (21%)	Bread (25%)	Pastry, cake and biscuits (28%)	Pastry, cake and biscuits (24%)	Pastry, cake and biscuits (18%)	Bread (23%)	Milk and milk products (21%)	Bread (26%)	Milk and milk products (17.%)	Pastry, cake and biscuits (21%)	Foods for specific dietetic use (21%)	Milk and milk products (12%)	Milk and milk products (12%)	Milk and milk products (20%)	Cheese (14%)	Pastry, cake and biscuits (13%)	Beverages (13%)
Milk and milk products (17%)	Cheese (20%)	Cheese (14%)	Beverages (17%)	Beverages (10%)	Beverages (13%)	Milk and milk products (16%)	Cheese (20%)	Meat, meat products and poultry (16%)	Cheese (10%)	Nuts, seeds and snacks (14%)	Pastry, cake and biscuits (13%)	Bread (11%)	Fish (9%)	Vegetables (7%)	Pastry, cake and biscuits (13%)	Nuts, seeds and snacks (12%)	Pastry, cake and biscuits (13%)
Meat, meat products and poultry (5%)	Eggs (4%)	Meat, meat products and poultry (9%)	Bread (9%)	Nuts, seeds and snacks (5%)	Foods for specific dietetic use (10%)	Cheese (11%)	Meat, meat products and poultry (11%)	Cheese (13%)	Nuts, seeds and snacks (10%)	Cheese (7%)	Beverages (8%)	Fish (10%)	Potatoes (8%)	Potatoes (7%)	Nuts, seeds and snacks (12%)	Cheese (10%)	Foods for specific dietetic use (12%)
Eggs (4%)	Cereal products (4%)	Eggs (5%)	Cheese (5%)	Fruit (3%)	Sugar, sweet fillings and sweet sauces (6%)	Fish (7%)	Fish (5%)	Soups (4%)	Beverages (9%)	Beverages (6%)	Fruit (7%)	Cheese (6%)	Vegetables (7%)	Fish (6%)	Beverages (8%)	Beverages (6%)	Fish (3%)

## 4. Discussion

The present study showed that the habitual dietary protein intake varies between elderly ranging from an average of 71 g/day in community-dwelling and frail to an average of 58 g/day in institutionalized elderly people, accounting for 16% and 17% of their energy intake, respectively. More than 80% of daily protein intake was distributed over the main meals, ranging between 10 and 12 g at breakfast, 15–23 g at lunch and 24–31 g at dinner. Plant-based protein contributed >50% of the protein intake at breakfast and 34%–37% at lunch, with bread as the dominant source. During dinner, >70% of the protein intake originated from animal protein.

Adequate dietary protein intake is a key factor for maintaining skeletal muscle mass in the elderly. The amount of protein intake, the distribution and the source of protein intake are all important to maximally stimulate postprandial muscle protein synthetic response and muscle mass accretion in the elderly [22]. We observed that habitual dietary protein intake is the lowest in the institutionalized elderly. Although the average protein intake of 0.8 g/kg-bw/day reaches the recommended daily allowance, a large proportion of institutionalized elderly people reported an insufficient protein intake below the estimated average protein requirement of 0.7 g/kg-bw/day [23]. Therefore, institutionalized elderly people are an important target population for nutritional intervention. Recent consensus statements have argued that protein intakes between 1.0 and 1.5 g/kg-bw/day may be necessary to slow down or counteract sarcopenia in the elderly [22,24,25,26]. The latter case suggests that not only institutionalized elderly but also community-dwelling and frail elderly people may need to improve their dietary protein intake. Protein intake in the elderly may be a result of the amount of food ingested. Indeed, we observed a strong positive correlation between total daily energy intake and protein consumption (Figure 1; *r* > 0.7, *p* < 0.001). This clearly shows that daily energy consumption is likely the most important factor driving protein consumption. In agreement, we did not observe any differences in macronutrient composition of diets between the various older subpopulations, which indicates that increasing protein consumption in the elderly is only feasible by either increasing total food intake or specific protein supplementation with (more) protein-dense foods or clinical nutritional supplements.

Daily protein intake was distributed over breakfast (15%–21%), lunch (26%–32%) dinner (38%–44%) and snacks (14.6%–18.3%). This protein distribution is similar to data from the US showing a skewed protein intake pattern [22,27]. Several studies suggest that a skewed protein intake pattern is less potent to stimulate muscle protein synthesis as opposed to protein intake which is evenly distributed [28,29]. In addition, 25–30 g of dietary protein per meal is suggested to maximally stimulate skeletal muscle protein synthesis in the elderly [22,27]. Ingestion of smaller, meal-like amounts of dietary protein attenuated the skeletal muscle protein synthetic response in elderly people when compared with young individuals [12,13]. In our study, we observed average protein intakes less than 12 g at breakfast. The latter protein intake is substantially below the proposed minimum of 20 g. Increasing protein intake at breakfast and lunch during exercise stimulated muscle protein synthesis [28,29] and augmented muscle mass gain in frail elderly people [16]. Therefore, increasing the amount of dietary protein at breakfast to at least 20 g might represent a promising dietary strategy to prevent or treat sarcopenia in elderly people.

Searching for strategies to further optimize muscle mass accretion in the elderly, we extended our research by evaluating the intake of specific protein sources as well as the timing of intake of those protein sources in the elderly. Evaluating the intake of specific protein sources is important in the elderly, as not only the quantity but also the quality of proteins are important to stimulate muscle protein synthesis and muscle mass gain [9,14,22]. A high quality protein largely depends on its digestibility and absorption kinetics as well as on the amino acid composition [23,30]. The digestibility and absorption of animal protein sources is between 90% and 99% [23]. Plant-based sources, however, are less digestible and varies between 70% and 90% [23]. In addition, whereas animal protein sources contain all amino acids in relatively the same amounts as the human body requires, plant-based protein may have insufficient amounts of amino acids to support bodily functioning [23,31]. In the community-dwelling, frail and institutionalized elderly, the majority of daily protein intake originated from animal protein sources (Figure 1). During breakfast, however, the majority of protein intake originates from plant-based protein sources in community-dwelling and frail elderly people (Table 2). Also at lunch, a large proportion of protein intake derives from plant-based protein sources with bread as the major source (Table 3). Bread may be considered a low-quality protein source as wheat and oats have a low protein-digestibility-corrected amino acid score (PDCAAS) [23,32]. Wheat provides limited quantities of isoleucine and lysine [23] and lower quantities of leucine and methionine as compared to animal type protein sources. This low quantity of essential amino acids may lead to an attenuated skeletal muscle protein synthetic response and loss of muscle mass in community-dwelling and frail elderly people [33]. In institutionalized elderly, the majority of dietary protein at breakfast and lunch originates from animal sources. Even though the relative intake (in %) from animal protein sources is high, the absolute intake (in g) from animal sources is still limited. The latter results in an intake of amino acids that is inadequate to augment muscle mass gain in institutionalized elderly people [12,13].

Despite the limited availability of some essential amino acids in plant-based proteins, a blend of complementary plant-based protein sources or a combination of plant- and animal-based protein should provide all essential amino acids [23,31]. Observing the data, all meals contain a blend of various plant-based and animal-based protein sources, which may limit the deficiency of essential amino acids. Still, the absolute amount of both plant- and/or animal-based protein at breakfast and lunch is inadequate to maximally stimulate muscle protein synthesis and muscle mass gain in the elderly. Therefore, increasing protein intake at breakfast and lunch with high-quality protein sources, which may come from animal- or plant-based protein sources, remains a promising strategy to postpone or treat sarcopenia.

## 5. Conclusions

Daily protein intake in these older populations is mainly (>80%) provided by the three main meals, with the most protein consumed during dinner. More than 60% of daily protein intake consumed is of animal origin, with plant-based protein sources representing nearly 40% of total protein consumed. During dinner, >70% of the protein intake originated from animal protein, while during breakfast and lunch a large proportion of protein is derived from-plant based protein sources.
